# Evidence on the State of the Medical Profession

**Published:** 1834-10-01

**Authors:** 


### INTELLIGENCE.

A PART of the evidence of the Physicians examined by the Committee appointed to inquire into the State of the Medical Profession is now before us, and we think it probable that our readers will be gratified by some extracts. When the whole of the evidence is printed, we shall endeavour to give an abstract of it.

### INCOME OF THE COLLEGE OF PHYSICIANS.

267. By a return laid on the table of this Committee from the College of Physicians, it appears that the income of the college for the year ending July 1831, was 1,099*l.* 17*s.* 9*d.*; for the year ending July 1832, 1,203*l.* 18*s.* 11*d.*; and for the year ending July 1833, 1,072*l.* 14*s.* 1*d.*; in the three instances, including all the sums receivable by the college on every different account. The same return states the expenditure of the college for the three consecutive years. Will you state to the Committee what is the pecuniary interest which the individual fellows of the college have, or can have, in the funds of the institution to which they belong?—They can have none whatever. It is rather a matter of expense than otherwise to the fellows.

268. What is the highest salary receivable by any fellow of the college from the funds of the institution?—The highest that is received is on a particular lecture; I think that is 32*l.* a year; the Lumlian lecturer gets 32*l.* a year. That was a benefaction and a stipulation, certain conditions stipulated by the person who left it for that purpose, to have a lecture given.

269. From the return which has been transmitted from the college, it appears that, in the two first years, the larger part of the stated income of the college has arisen from the rents of lands and houses; and in the third year, the proportion of 437*l.* out of 1,072*l.* From what sources is the permanent income derived, to be described as rents of lands and houses?—There are two small estates, one left by Dr. Harvey, the discoverer of the circulation of the blood, and one by Dr. Hamey, and a house, inhabited by Linane. In the general deterioration in the value of land, the rent has fallen very considerably.

270. Both of those are benefactions from former fellows of the college?—Yes.

271. Of the funds so left to the college by former fellows, increased as they may be by the addition of fellows each year, you have stated that the largest sum received by any fellow is the sum of 32*l.* Can you state to the Committee what is the number of fellows who receive any pecuniary benefit from the college, and what is the average of the sums which such fellows receive from the college?—The utmost number of those who receive any thing from the college at all, is eleven; and the average of their receipt is 22*l.*

272. The funds of the college, of which a statement has been submitted to the Committee, contain sundry items for the admission of fellows: the sums by which the college is supported are partly derived from the contributions of the existing members?—Exactly so.

273. No object, therefore, of a pecuniary nature can be supposed to weigh with the members of the college?—Probably not; as it is a fact, that the members of the college have contributed 8,000*l.* towards the building their present college.

274. You have stated that the funds of the body are derived from the benefactions of former fellows, or contributions of living fellows in a great measure?—Precisely so.

275. With the proportion in the first year of 180*l.* 10*s.*, in the second year of 252*l.* 14*s.*, and in the third year of 324*l.* 14*s.* for the admission of licentiates, are the Committee to understand that the fees for admission of licentiates include the fees paid by those who are admitted to the two English Universities, as well as the candidates who are educated elsewhere, and who are equally eligible to practise physic on the payment of such fee and the obtaining of such licence?—There is a difference between what a fellow pays and what a licentiate pays.

275. What do the censors receive for the discharge of their duty?—20*l.* a year a-piece.

277. In the return made by the college as to the patronage which the college exercises, there is no notice taken of their power, in case of a vacancy, to recommend a physician to St. Bartholomew's Hospital, on Dr. Hamey's foundation: is that power still exercised by the College?—It is still exercised.

278. That is an accidental omission?—It is.

279. You have stated that the college is not able to exclude irregular practitioners from practice, and that the college have abandoned their attempts to put down such practitioners by legal proceedings?—Not altogether; for it is within three years a prosecution was instituted which cost the college 200*l.*

280. It failed?—Yes; for the defendant pleaded, at last, that he had been only practising as a surgeon.—*Sir Henry Halford's Evidence.*

### DISQUALIFICATIONS FOR THE FELLOWSHIP.

1018. Ought the fellows and licentiates of the college, in your opinion, to be allowed to practise midwifery?—The licentiates practise midwifery, the fellows do not.

1019. Is the restriction proper?—It has always been my opinion that it is not proper; but the opinion of others who have had a vast deal more experience in the profession than myself, is opposed to that.

1020. It appears that, according to the present bye-laws, the following persons are disqualified from becoming candidates or fellows of the college: first, any one that has used any nostrum in curing diseases for gain?—Certainly; a fellow who employs a nostrum is liable to expulsion.

1021. Second, any one who has ever gained a living by practising as an apothecary?—That law has been modified: there is such a law, but there is an exception made in the law by introducing the words, "sine gravi aliqua de causâ," which takes all the violence out of it. This qualification existed in the Universities, and it was intended to prevent a man practising as an apothecary in the University from having his name upon the boards of a college, and proceeding to take a doctor's degree; and in London the same thing might prevail. A man might be keeping a shop and attending patients, and at the end of a short time, by means of a qualification, he would become a physician. I think that is a sort of underselling which is injurious to the profession, and injurious to education.

1022. The same law extends to a person who has gained a living by practising midwifery: is that a restriction you approve of?—It is not.

1023. Fourth, any one who has gained a living by selling any merchandize; the words are, "aut mercibus quibusvis vendendis victum quæsitaverit." What interpretation do you put upon them."—A dealer.

1024. A shopkeeper or a petty dealer?—That is my idea.

1025. You do not think that a merchant, who thought proper afterwards to enter medicine, might under this statute be disqualified?—I have no idea of it; but I do not contemplate such a case.

1026. May not possibly the word "merces" bear the interpretation of apothecaries' wares?---Very probably.

1027. Is that the interpretation you would be disposed to give to it?---To speak the truth, I did not know that that portion of the law existed.

1028. After a person has been admitted a candidate, or a fellow, does he not absolutely forfeit his seat, if he shall gain a living by practising as an apothecary or midwife, or "mercibus quibusvis vendendis?"---A man may be elected a fellow who has practised pharmacy; a man may be elected a fellow who has practised midwifery; but he must cease to practise them upon being elected a fellow or a candidate.

1029. Or else there is an absolute forfeiture?---Yes.

1030. Then if he has been guilty, after being elected candidate or fellow, of selling any nostrum, he is liable to be expelled from the college, if the majority of the fellows assembled in the comitia majora think proper to decide so by ballot?---Clearly.

1031. And if he has been convicted criminis alicujus gravioris ac publici, he is in the same manner liable to be expelled by ballot at the comitia majora?—Clearly.

1032. It appears that in the case of his practising as an apothecary or a midwife, or selling the merces aliquas, he absolutely forfeits his seat; whereas, if he sells a nostrum, or is convicted of some grievous crime, the college deliberate upon his offence; there is therefore a heavier visitation upon him for practising as an apothecary or midwife, than for being found guilty criminis alicujus gravioris ac publici: does not that seem to be an anomaly in the statutes?—I have already mentioned that, with reference to midwifery, in my opinion the law is injurious; but, with regard to the other, it appears to me that the statute means to say, that if he shall have been convicted of some crime, yet still a possibility might arise on the part of the college that they might think he had been improperly convicted of such a crime.

1033. To your knowledge, do any candidate or inceptor candidates now practise midwifery?—I think there is an inceptor candidate practising midwifery.

1034. Under the statute, the inceptor candidates are not subject to any penalty?—I believe not; but I apprehend it would be construed as applicable to an inceptor candidate. It might be an objection upon a ballot: it was an objection in Dr. Lett's case, if I remember right.—*Dr. Seymour's Evidence.*

